# Harm-reduction goals and safer-drinking strategies among individuals attending a new drop-in center

**DOI:** 10.1186/1940-0640-10-S2-O19

**Published:** 2015-09-24

**Authors:** Véronique S Grazioli, SE Collins, S Paroz, C Graap, J-B Daeppen

**Affiliations:** 1 Department of Community Medicine and Health, Alcohol Treatment Center, Lausanne University Hospital, Lausanne, Switzerland; 2 Department of Psychiatry and Behavioral Sciences, Harborview Medical Center, University of Washington, Seattle, WA, USA

## Background

Although socially marginalized individuals with alcohol-use disorders (AUDs) experience severe alcohol-related harm, few enter treatment. [1,2] Developing innovative, tailored interventions is therefore important to addressing this population needs. The Harm Reduction Treatment - Brief Intervention (HaRT-BI), designed to elicit self-generated harm-reduction goals and discuss safer-drinking strategies [3], was adopted for a new drop-in center that allows drinking in Switzerland. This study aimed to qualitatively document participants' self-generated harm-reduction goals and safer-drinking strategies endorsement at the HaRT-BI baseline session.

## Material and methods

Participants (N = 78; 16.7% female; mean age = 38) were socially marginalized individuals with AUDs participating in a larger study evaluating a new facility attendance and subsequent drinking outcomes. At baseline, study interventionists elicited participants' harm-reduction goals with an open-ended question (‘What would you like to see happen for you in the next 4 weeks?’) and provided participants with a list of 12 safer-drinking strategies (e.g., taking vitamins, counting drinks). (3) Content analysis was used to categorize the goals and strategies participants endorsed.

## Results

Seventy-six participants (97.4%) generated and endorsed at least one goal (*Mdn* = 2.1, *IQR* = 2) and one strategy (*Mdn* = 3, *IQR* = 1). The 5 most highly endorsed goals categories included drug and alcohol-related goals (e.g., reducing, connecting with treatment), basic-need goals (i.e., searching housing), health-related goals (i.e., improving health), and quality-of-life goals (i.e., engaging in meaningful activities). Changing manners of drinking (e.g., spacing drinks) was the most highly endorsed strategy type, followed by buffering the effects of alcohol on the body (e.g., eating) and reducing drinking.

## Conclusions

Most participants did generate and endorse harm-reduction goals and safer-drinking strategies, which replicated US findings [4,5]. These results suggest that HaRT-BI may be used to help these individuals set harm-reduction goals and safer-drinking strategies. Future research is needed to test HaRT-BI effectiveness in decreasing alcohol outcomes.
